# *Brucella ovis* mutant in ABC transporter protects against *Brucella canis* infection in mice and it is safe for dogs

**DOI:** 10.1371/journal.pone.0231893

**Published:** 2020-04-16

**Authors:** Camila Eckstein, Juliana P. S. Mol, Fabíola B. Costa, Philipe P. Nunes, Pâmela A. Lima, Marília M. Melo, Thaynara P. Carvalho, Daniel O. Santos, Monique F. Silva, Tatiane F. Carvalho, Luciana F. Costa, Otoni A. O. Melo Júnior, Rodolfo C. Giunchette, Tatiane A. Paixão, Renato L. Santos

**Affiliations:** 1 Departamento de Clínica e Cirurgia Veterinárias, Escola de Veterinária, Universidade Federal de Minas Gerais, Belo Horizonte, Minas Gerais, Brazil; 2 Departamento de Patologia Geral, Instituto de Ciências Biológicas, Universidade Federal de Minas Gerais, Belo Horizonte, Minas Gerais, Brazil; 3 Departamento de Morfologia, Instituto de Ciências Biológicas, Universidade Federal de Minas Gerais, Belo Horizonte, Minas Gerais, Brazil; East Carolina University Brody School of Medicine, UNITED STATES

## Abstract

**Background/Objectives:**

Vaccination is the most important tool for controlling brucellosis, but currently there is no vaccine available for canine brucellosis, which is a zoonotic disease of worldwide distribution caused by *Brucella canis*. This study aimed to evaluate protection and immune response induced by *Brucella ovis* Δ*abcBA* (*Bo*Δ*abcBA*) encapsulated with alginate against the challenge with *Brucella canis* in mice and to assess the safety of this strain for dogs.

**Methods:**

Intracellular growth of the vaccine strain *Bo*Δ*abcBA* was assessed in canine and ovine macrophages. Protection induced by *Bo*Δ*abcBA* against virulent *Brucella canis* was evaluated in the mouse model. Safety of the vaccine strain *Bo*Δ*abcBA* was assessed in experimentally inoculated dogs.

**Results:**

Wild type *B*. *ovis* and *B*. *canis* had similar internalization and intracellular multiplication profiles in both canine and ovine macrophages. The *Bo*Δ*abcBA* strain had an attenuated phenotype in both canine and ovine macrophages. Immunization of BALB/c mice with alginate-encapsulated *Bo*Δ*abcBA* (10^8^ CFU) induced lymphocyte proliferation, production of IL-10 and IFN-γ, and protected against experimental challenge with *B*. *canis*. Dogs immunized with alginate-encapsulated *Bo*Δ*abcBA* (10^9^ CFU) seroconverted, and had no hematologic, biochemical or clinical changes. Furthermore, *Bo*Δ*abcBA* was not detected by isolation or PCR performed using blood, semen, urine samples or vaginal swabs at any time point over the course of this study. *Bo*Δ*abcBA* was isolated from lymph nodes near to the site of inoculation in two dogs at 22 weeks post immunization.

**Conclusion:**

Encapsulated *Bo*Δ*abcBA* protected mice against experimental *B*. *canis* infection, and it is safe for dogs. Therefore, *B*. *ovis* Δ*abcBA* has potential as a vaccine candidate for canine brucellosis prevention.

## Introduction

Canine brucellosis is a zoonotic disease caused by *Brucella canis* [1]. In dogs, infection is associated with reproductive disease characterized by outbreaks of abortion, conception failure, or epididymitis and orchitis in males [2,3]. Although human brucellosis due to *B*. *canis* is considered infrequent and less pathogenic when compared to other *Brucella* species [4], the close contact between dogs and humans makes the zoonotic risk posed by *B*. *canis* highly significant under a public health perspective [5]. *B*. *canis* infections have been diagnosed worldwide [5]. In the Americas, Asia and Africa, canine brucellosis is considered endemic in dogs [6]. Although in certain countries such as the United Kingdom [7] and Sweden [8], *B*. *canis* infection in dogs is less frequent.

In contrast to well-established serological methods for diagnosing smooth *Brucella* infections, including *B*. *melitensis*, *B*. *suis* and *B*. *abortus*, which commonly affect farm animals, serologic diagnosis of canine brucellosis remains a challenge [9–13]. Definitive diagnosis is based on bacterial isolation from biological samples including blood, placenta, semen, urine, and vaginal swabs [2,14–16]. However, *B*. *canis* isolation requires appropriate laboratory conditions, it is expensive and time consuming, with high possibilities of false-negative results due to the intermittent shedding of *B*. *canis*. In addition, treatment of infected dogs with antibiotics is not encouraged due to the high rate of relapse and uncertain results [17,18]. Although euthanasia of infected dogs has been considered as an alternative to reduce the prevalence of the disease [3], an efficient control of canine brucellosis should be based on vaccination of dogs, which would reduce the risk of transmission for humans [5]. Currently, there is no vaccine available for controlling canine brucellosis, whereas vaccines applied to livestock have residual virulence and are not indicated to dogs.

ABC transporters are considered virulence factors of *Brucella* spp. [19,20]. *B*. *ovis* has a specific ABC transporter encoded by the *B*. *ovis* pathogenicity island 1 (BOPI-1), which is required for pathogenesis [20,21]. Absence of this particular ABC transporter results in attenuation *in vitro* and *in vivo* in mice and sheep [20,22–24], although the strain remains immunogenic in rams [22], and when used as experimental vaccine resulted in sterile immunity against *B*. *ovis* experimental infections in rams [25]. Importantly, among vaccination strategies for controlling brucellosis, the use of mutant and attenuated strains induces higher protection indexes when compared to other vaccine categories in the mouse model [26].

Considering that *B*. *ovis* is not pathogenic for humans and dogs as well as the structural similarities between *B*. *canis* and *B*. *ovis*, which have a naturally rough LPS [27], this study aimed to evaluate the protective capacity of the *B*. *ovis* vaccine candidate strain (*B*. *ovis* Δ*abcBA*–with deletion of *abcA* and *abcB* genes from the BOPI-1) against *B*. *canis* infection in mice and to evaluate the safety of this vaccine strain in dogs.

## Material and methods

### Ethics statement

Animal experiments followed all applicable laws and regulations and experimental protocols were approved by the institutional Ethics Committee on Animal Use (CEUA-UFMG, Protocols: 244/2014 and 329/2017). Mice were euthanized with 2% xylazine hydrochloride (0.6 mg/kg) and 1% ketamine hydrochloride (27 mg/kg) intraperitoneally, followed by cervical dislocation. Immunized dogs were submitted to the euthanasia by a veterinarian, using thiopental sodium (35 mg/kg, iv) followed by intravenous injection of a saturated solution of potassium chloride.

### Bacterial strains and culture conditions

*B*. *canis* ATCC 23365, *B*. *ovis* ATCC 25840, and *Bo*Δ*abcBA* [20] were used in this study. *B*. *ovis* ATCC 25840 and *Bo*Δ*abcBA* were grown on tryptose soy agar (TSA) with 1% hemoglobin (Becton Dickinson, Brazil), for 3 days at 37°C in a humidified 5% CO_2_ atmosphere. To identify *Bo*Δ*abcBA*, kanamycin (100 μg/mL, Gibco, Brazil) was added to the culture medium when needed. Bacteria were suspended in sterile PBS (phosphate buffer saline, pH 7.4, Sigma-Aldrich, USA), and inocula concentrations were estimated by spectrophotometry (SmartSpec Plus Bio-Rad) at an optical density of 600 nm (OD_600_). *B*. *canis* was grown in tryptose soy broth (TSB) overnight (16–18 h) under agitation (150 rpm) at 37°C, followed by centrifugation at 2,000 x *g* for 10 min at 21°C. The pellet was resuspended in sterile PBS. *B*. *canis* culture was performed under biosafety level 3 conditions. Inactivated bacterial suspensions were prepared using gamma irradiation.

### Ovine and canine monocyte-derived macrophages isolation, culture, and infection

Dogs from an institutional vivarium and sheep obtained from a commercial source were used in this experiment. These animals were considered free of *Brucella* spp. as determined by agar gel immunodiffusion (AGID) and blood PCR [28].

Monocyte-derived macrophages were obtained as described [24]. Ovine and canine macrophages were inoculated with *B*. *ovis*, *B*. *canis* or *Bo*Δ*abcBA* at a multiplicity of infection (MOI) of 100. Plates were centrifuged at 400 x *g* for 5 min at 21°C, and then incubated for 30 min at 37°C in 5% of CO_2_. Inocula were removed, and cells washed with sterile PBS and incubated with gentamicin (50 μg/mL) diluted in RPMI for 1 hour. Cells were then washed once and incubated with sterile water for 20 min for lysis. Cells were mechanically removed and each well was washed with sterile PBS, and serially diluted and plated on TSA with 1% of hemoglobin with or without kanamycin, incubated for 3 to 5 days at 37°C and 5% CO_2_ for CFU counting. Similar procedures were repeated at 4, 24, and 48 h post infection (hpi) to estimate intracellular multiplication of bacteria. After removal of RPMI with 50 μg/mL of gentamicin, cells were maintained in RPMI medium containing 25 μg/mL of gentamicin, until lysis. Three independent experiments were performed in triplicates.

### Lymphocyte proliferation assay

Three groups of 6 to 7-week-old BALB/c mice were subcutaneously immunized with 100 μL of alginate-encapsulated *Bo*Δ*abcBA* (10^8^ CFU per mouse) (n = 7), sterile alginate capsules (n = 7) or PBS (n = 6). Spleens were aseptically collected at 4 weeks after immunization and macerated in a sterile Petri dish with a syringe plunger. Cells were transferred to a conical tube, homogenized with a pipette and centrifuged at 300 x *g* for 10 min at 4°C. Cells were resuspended with red blood cell lysis buffer (ammonium tris-chloride), incubated for 5 min and centrifuged at 300 x *g* for 10 min at 4°C. Cells were then washed three times with sterile PBS followed by centrifugation (300 x *g* for 10 min at 4°C) and resuspended in RPMI with 10% FBS.

Cell viability was evaluated by trypan blue exclusion. Cells were seeded in duplicates for each treatment in 96-well-plate containing 5 x 10^5^ viable cells per well in 100 μL. Cells were stimulated with 100 μL of RPMI (negative control), or *B*. *canis* or *B*. *ovis* inactivated by gamma irradiation (equivalent to 10^8^ CFU/well). Plates were incubated at 37°C with 5% of CO_2_ for 72 h. Then, plates were centrifuged at 400 x *g* for 10 min and supernatants were collected and stored at -20°C for cytokine measurement.

For assessment of lymphocyte proliferation, 20 μL of 5 mg/mL MTT reagent (3-(4,5-dimethylthiazol-2-yl)-2,5-diphenyltetrazolium bromide) (Invitrogen, USA) was added to each well and incubated for 2 h at 37°C and 5% of CO_2_, protected from light, followed by addiction of 100 μL of 10% SDS (sodium dodecyl sulfate) with HCl. After incubation overnight at 37°C and 5% of CO_2_ plates were read in an ELISA reader at 596 nm.

### Cytokine responses

Concentrations of IFN-γ and IL-10 in supernatant of stimulated splenocytes were measured by sandwich ELISA (DuoSet ELISA, R&D Systems, USA) according to the manufacturer’s instructions.

### *Brucella ovis* Δ*abcBA* encapsulation and protection assay in mice

*Bo*Δ*abcBA* encapsulation was performed as previously described [29]. Mice were purchased from UFMG Central Bioterium, kept at 22–24°C with cycles of 12-hours of light/darkness, with food and water *ad libitum*. Two groups of female, 10 to 12-week-old BALB/c mice were subcutaneously immunized with a single dose of 100 μL of 10^8^ CFU of alginate-encapsulated *Bo*Δ*abcBA* (n = 5) or inoculated with the same volume of PBS (n = 5). Four weeks after immunization, mice were challenge intraperitoneally with 10^6^ CFU/mice of *B*. *canis*.

Two weeks after the challenge, spleen homogenates were plated for CFU counting. Briefly, Spleen of mice were weighed and homogenized in 2 mL of sterile PBS with a mixer (Hamilton Beach, USA). Homogenates were serially (10-fold) diluted in sterile PBS, and plated in duplicates on TSA with 1% hemoglobin. CFU counting was done after 3 days of incubation at 37°C and 5% CO_2_. To differentiate vaccine (*Bo*Δ*abcBA*) and challenge strains, diluted homogenates were plated on TSA with 1% hemoglobin and 100 μg/mL kanamycin.

Fragments of spleen and liver were submitted to histopathology and immunohistochemistry. Protection indexes were determined considering the difference in log of CFU in the spleens of non-vaccinated controls and vaccinated mice.

### Immunization of dogs

Ten mix-breed 1 to 2 year-old dogs (5 females and 5 males) were tested twice for detection of *B*. *canis* infection by clinical evaluation, bacterial isolation and PCR for *Brucella* spp. [29], using blood, semen, urine and vaginal swabs. The dogs were also serologically tested by AGID, and divided in two groups: one inoculated with sterile alginate capsules (n = 5; 3 females and 2 males), and the other immunized with alginate-encapsulated *Bo*Δ*abcBA* (n = 5; 2 females and 3 males).

Dogs were subcutaneously inoculated with 1 mL of sterile capsules or with alginate encapsulated *Bo*Δ*abcBA* (1 x 10^9^ CFU). After immunization, dogs were clinically evaluated at 1, 2, 3, 5, and 9 days, and then every two weeks after immunization. Blood and urine samples, and vaginal swabs from females or semen samples from males were collected every two weeks after immunization, and submitted to hematological analysis, bacterial isolation and PCR. Alertness, appetite, weight, local swollen at the site of injection (measured with cutimeter), rectal temperature, and changes in volume and consistency of superficial lymph nodes (mandibular, superficial cervical, inguinal, and popliteal) were recorded. The reproductive system was evaluated by observing vaginal secretions in females, inspection and palpation of the testes and epididymis in males [30].

Serum was collected and stored at -20°C. Blood samples collected in sodium citrate were submitted to hematological analysis, bacterial isolation, and PCR. Two vaginal swabs were obtained from each female, resuspended in 1 mL of sterile PBS, and then processed for DNA extraction and bacterial isolation. Semen samples were collected directly in sterile conical tubes of 15 mL as described [30]. Urine samples were collected using sterile catheters into 15 mL sterile tubes, concentrated by centrifugation at 4,000 x *g* for 10 min, resuspended in 2 mL of supernatant urine, and then submitted to DNA extraction and bacterial isolation.

Twenty-one weeks after immunization, dogs immunized with alginate-encapsulated *Bo*Δ*abcBA* were submitted to the euthanasia and necropsy. Samples of skin (at the site of immunization), lymph nodes (retromandibular, axillar, inguinal, popliteal, subscapular, and cervical), spleen, liver, pancreas, stomach, urinary bladder, kidneys, urethra, ureter, testicles/ovaries, epididymides, prostate/uterus, penis, central nervous system, heart, lung, and bone marrow were collected into sterile tubes containing 2 mL of PBS, homogenized, and submitted to bacterial isolation and PCR. Samples from those organs were also processed to histopathology.

For bacterial isolation, prior to immunization, blood, urine, vaginal swabs or semen from dogs were inoculated on Tryptose agar (BD Difco, USA) without or with selective supplementation (2,500 UI of polymyxin B; 12,500 UI bacitracin; 50,000 UI of nystatin; 50 mg of cycloheximide; 2.5 mg of nalidixic acid; and 10 mg of vancomycin) (Sigma Aldrich, USA), and in 10 mL of tryptose broth (BD Difco, USA) containing selective supplement. Cultures were incubated at 37°C with 5% CO_2_, and bacterial colony growth was checked every 48 h for 21 days. After 7 days in culture, 100 μL of broth were plated on tryptose agar, and kept at 37°C with 5% CO_2_ for 21 days. Bacterial isolation over the course of the experiment was performed using 100 μL of whole blood, semen, urine, or vaginal swab homogenates that were plated on Thayer Martin agar with 1% of hemoglobin, with or without kanamycin (100 μg/mL). Plates were incubated at 37°C with 5% CO_2_ for 21 days.

Tissue samples were homogenized using a tissue homogenizer (CB, Biotech, Brazil), and plated on Thayer Martin agar with 1% of hemoglobin with or without kanamycin (100 μg/mL) and incubated at 37°C with 5% CO_2_ for 21 days. Bacterial colonies were heat-killed (100°C for 30 min) and submitted to PCR for confirmation.

### Serology

Serologic analysis was performed using a commercial AGID kit (TECPAR, Brazil), according to the manufacturer’s instructions.

### DNA extraction and Polymerase Chain Reaction (PCR)

DNA extraction from semen and urine was performed using the proteinase K and phenol/chloroform method, with 500 μL and 1 mL, of semen and urine, respectively [31]. DNA extraction from blood and vaginal swabs was performed using the guanidine method [32], with 250 μL of each sample.

All samples were submitted to a generic PCR [31] targeting the *bcsp31* gene of *Brucella* spp. To identify the vaccine strain, positive samples that were positive in the generic PCR to *Brucella* spp. were submitted to a species-specific PCR to detect *B*. *ovis* [33], followed by a PCR to detect the vaccine strain *Bo*Δ*abcBA* [25]. DNA extracted from pure cultures of *B*. *ovis* was used as positive control for general and specific detection of *B*. *ovis* or *Bo*Δ*abcBA*. In negative controls template DNA was replaced with water. All primers and expected products are summarized in S1 Table.

### Hematological and biochemical analysis

Hematological parameters were measured by an automatic analyzer (PocH-100iV—Sysmex, Japan). Commercially available kits (Kovalent, Brazil) were used to measure alanine aminotransferase (ALT), aspartate aminotransferase (AST), urea, creatinine, alkaline phosphatase, bilirubin, and alfa-amylase, and readings were performed using a spectrophotometer (Cobas Mira, Roche, Switzerland).

### Histopathology and immunohistochemistry

Tissues were fixed for 24 h immediately after euthanasia by immersion in 10% buffered formalin or Bouin’s solution in the case of testicles and epididymis, then processed for paraffin embedding. Four μm sections were stained with hematoxylin and eosin (HE). Immunohistochemistry (IHC). IHC was performed as described [34]. Briefly, 3–4 μm-thick sections were incubated with peroxidase blocking reagent (Kit EnVisionTM FLEX, Dako, USA) at 22°C for 30 min, rinsed in PBS (1.5 M NaCl, 0.1 M Na_2_HPO_4_, 0.01 M NaH_2_PO_4_), incubated with skim milk diluted in PBS (1 g/40 mL) for 40 min at 22°C, followed by incubation with a monoclonal anti-*B*. *melitensis* primary antibody (1:100 dilution) for 1 h. Sections were then incubated with the developing reagent (HRP-Kit EnVisionTM FLEX, Dako, USA) for 20 min at 22°C. Reaction was revealed with the 3, 3'diaminobenzidina (DAB) chromogen and counterstained with Meyer hematoxylin. The primary antibody was replaced with PBS as negative controls.

Histopathology slides of mouse liver and spleen were examined by a veterinary pathologist and blindly subjected to a scoring system ranging from 0–3 (0: absence of lesion; 1: mild; 2: moderate, and 3: severe) for microgranulomas in the spleen, and microgranulomas, necrosis, and thrombosis in the liver (resulting in a combined score up to 9).

### Statistical analysis

CFU data were logarithmically transformed and subjected to ANOVA. Means were compared by unpaired T student for protection assay in mice and Tukey test for *in vitro* macrophage assay. Histopathology scores were compared using the Kruskal-Wallis non-parametric test. Frequency of positive samples in AGID, bacterial isolation and PCR were compared by the Fisher’s exact test. Means of hematological and biochemical parameters as well as semen concentration, rectal temperature and swelling at the skin inoculation site were compared using the Sidak’s multiple comparisons test or Dunnett’s multiple comparisons test. Statistical analyses were performed using the GraphPad Prism 5.0 software (GraphPad, USA).

## Results

### *Brucella ovis* Δ*abcBA* is attenuated in primary canine macrophages

**Fig 1:**
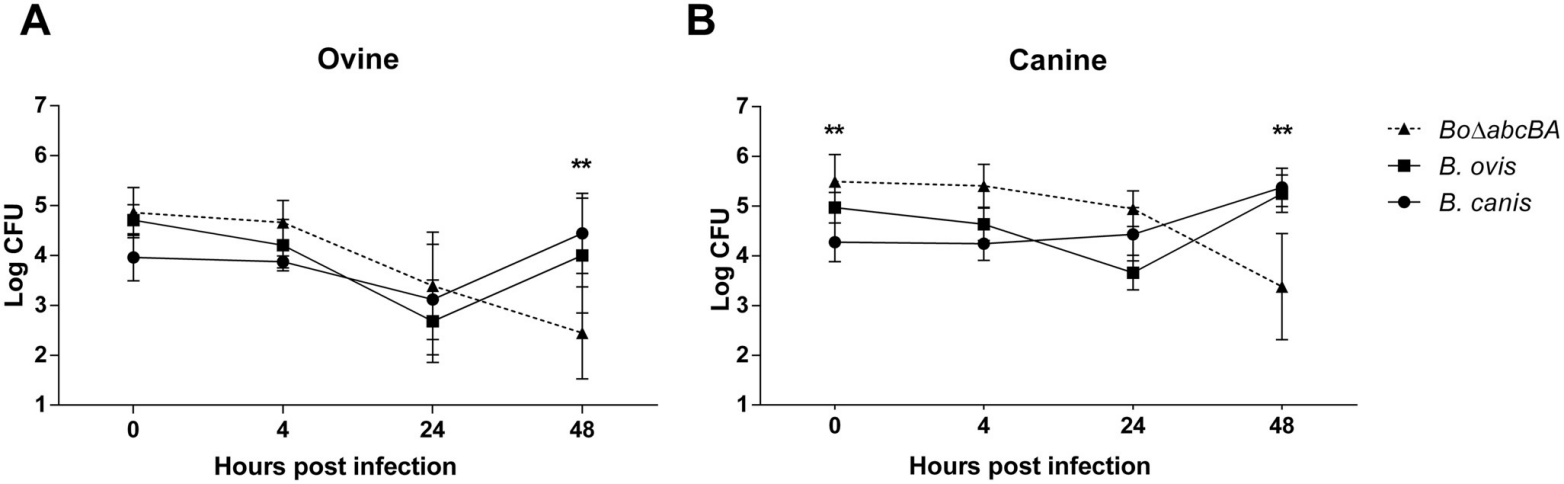
Internalization and multiplication of *B*. *ovis* Δ*abcBA*, wild type (WT) *B*. *ovis*, and WT *B*. *canis* in ovine (A) and canine (B) primary macrophages. Macrophages were inoculated with *B*. *ovis*, *B*. *canis* or *Bo*Δ*abcBA* at MOI of 100 and lysed at 0, 4, 24, and 48 hpi to estimate intracellular internalization and multiplication of bacteria. Data represent mean and standard deviation of three independent experiments. Data were submitted to ANOVA and means were compared by the Tukey test. Asterisk indicate statistically significant differences between the mutant strain *Bo*Δ*abcBA* and WT *B*. *canis* or *B*. *ovis* at each time point (**p < 0.01).

There are no reports *B*. *ovis* infections in dogs or studies that indicate the capacity of this species to infect dogs or canine cells *in vitro*. Assessment of intracellular attenuation of the candidate vaccine strain *Bo*Δ*abcBA* in canine macrophages is a preliminary parameter of safety, which is relevant to further evaluating this strain for prevention of canine brucellosis. To evaluate the potential for internalization and survival of *Bo*Δ*abcBA*, primary canine and ovine macrophages were infected with wild type (WT) *B*. *canis*, WT *B*. *ovis*, or *Bo*Δ*abcBA* (Fig 1).

*B*. *ovis* and *B*. *canis* had similar kinetics in canine and ovine macrophages. Both strains were stable or had a minor decline at 24 hpi, with evident intracellular multiplication at 48 hpi. In contrast, the mutant strain *Bo*Δ*abcBA* was not capable of intracellular multiplication in canine and ovine macrophages with significantly reduced intracellular CFU numbers (p < 0.01) at 48 hpi, indicating a strongly attenuated phenotype in both ovine and canine macrophages (Fig 1).

Interestingly, internalization of *Bo*Δ*abcBA* (0 hpi) in canine macrophages was significantly higher when compared to the WT *B*. *canis* strain (p < 0.01). However, this higher internalization rate did not influence the profile of attenuation and reduction of *Bo*Δ*abcBA* population inside canine macrophages at 48 hpi. Therefore, the *Bo*Δ*abcBA* mutant strain was capable of infecting ovine and canine primary macrophages, and this strain displayed an attenuated phenotype in macrophages from these two host species.

### Immune response induced by alginate-encapsulated *Brucella ovis* Δ*abcBA* in mice

**Fig 2:**
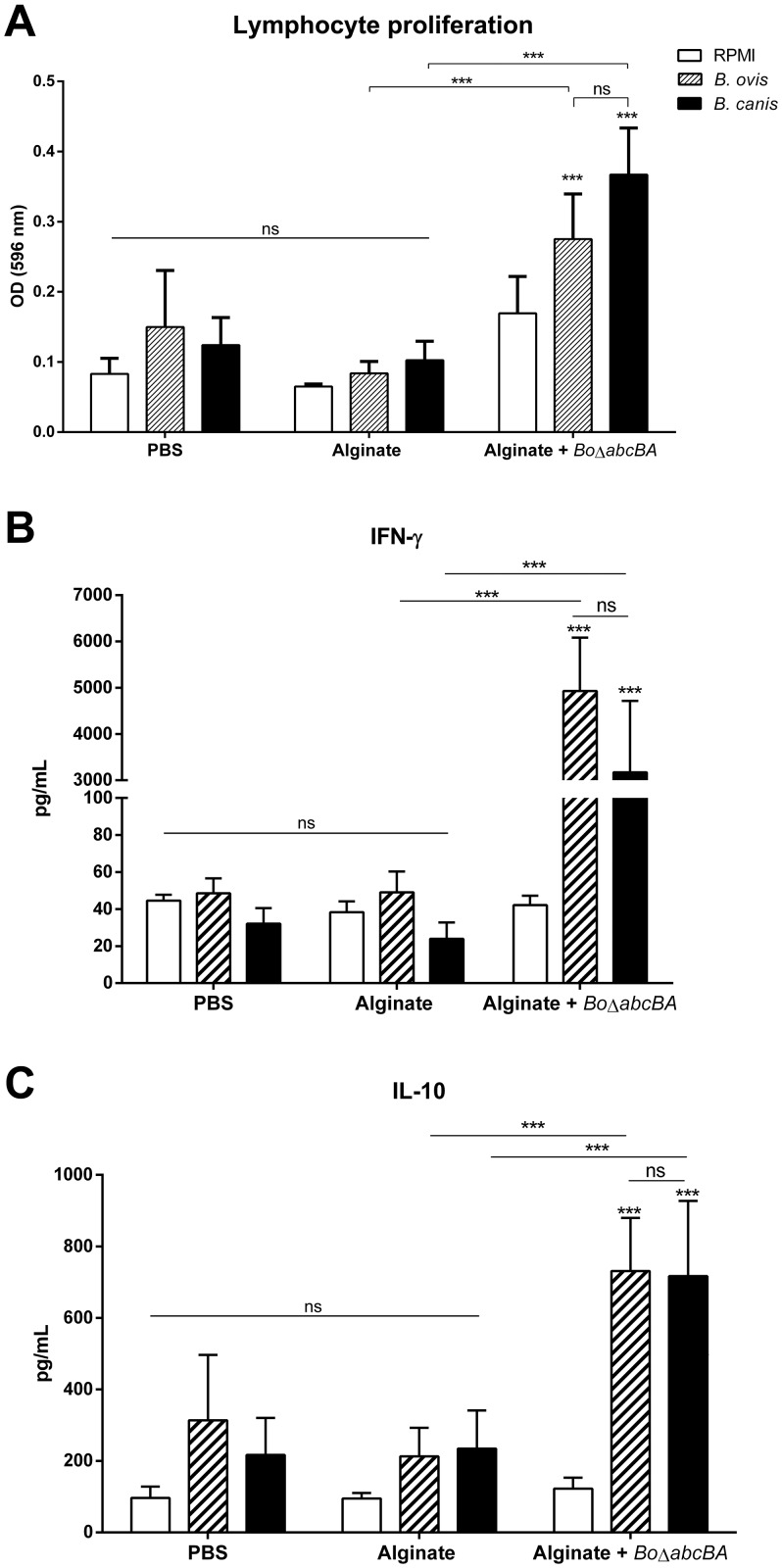
Lymphocyte proliferation assay (A), IFN-γ (B) and IL-10 (C) production by splenocytes from mice immunized with alginate-encapsulated *Brucella ovis* Δ*abcBA* (*Bo*Δ*abcBA*; n = 7) or mice inoculated with sterile alginate (n = 7) or PBS (n = 6). (A) After four weeks of immunization, splenocytes of mice (5 x 10^5^) were stimulated with RPMI, *B*. *ovis* or *B*. *canis* inactivated by gamma irradiation (corresponding to 10^8^ CFU/well) for 72 h and proliferative response were analyzed by MTT assay. Data correspond to the mean and SD at minimum of 6 mice for each group with duplicates. Data were submitted to ANOVA and means were compared using the Tukey test (***p < 0.001). (B and C) Four weeks after immunization, splenocytes were recovered and stimulated with RPMI (negative control), *B*. *ovis* or *Brucella canis* inactivated by gamma irradiation. At 72 h after stimulation, supernatants were recovered and levels of cytokines were evaluated by specific sandwich ELISA. Each data point represents mean and SD of 6 or 7 mice in duplicates for each stimulus. Data were submitted to ANOVA and means were compared using the Tukey test (***p < 0.001).

Internalization of *Bo*Δ*abcBA* by canine macrophages with a kinetic similar to that of ovine macrophages supports the notion that this candidate vaccine strain may result in antigen presentation. Therefore, we evaluated cellular immune responses promoted by *Bo*Δ*abcBA* upon stimulation with WT *B*. *canis*. Alginate encapsulation has provided better protection when compared to the non encapsulated vaccine strain [24]. Splenocytes from BALB/c mice immunized with alginate-encapsulated *Bo*Δ*abcBA* (n = 7) or non-immunized controls (capsules of sterile alginate n = 7 or PBS n = 6) were used in an *in vitro* cell proliferation assay at 4 weeks after immunization (Fig 2).

Significantly higher lymphocyte proliferation (p < 0.001) was observed in mice immunized with alginate-encapsulated *Bo*Δ*abcBA* when compared to mice inoculated with sterile alginate capsules or PBS. Importantly, stimulation of splenocytes from mice immunized with alginate-encapsulated *Bo*Δ*abcBA* with either γ-irradiated *B*. *ovis* or *B*. *canis* resulted in lymphocyte proliferation significantly higher when compared to splenocytes treated with RPMI (p < 0.001), and there was no significant difference between splenocytes stimulated with *B*. *ovis* or *B*. *canis* (p > 0.05) further supporting the potential of *Bo*Δ*abcBA* for inducing a protective immune response against *B*. *canis*.

To better characterize the immune responses, concentrations of IFN-γ and IL-10 were measured in supernatants of splenocytes from immunized and control mice as in the previous experiment, and stimulated with *B*. *ovis* or *B*. *canis* inactivated by gamma irradiation (Fig 2). Splenocytes from mice immunized with alginate-encapsulated *Bo*Δ*abcBA* and stimulated with *B*. *ovis* or *B*. *canis* inactivated by gamma irradiation responded producing high levels of IFN-γ and IL-10 when compared to splenocytes from mice inoculated with PBS or sterile alginate capsules (Fig 2).

Production of IFN-γ by splenocytes from mice immunized with alginate-encapsulated *Bo*Δ*abcBA* and stimulated with *B*. *ovis* inactivated by gamma irradiation was approximately 100-fold higher (p < 0.001) when compared to splenocytes stimulated with RPMI. Notably, similar results were observed in the group immunized with alginate-encapsulated *Bo*Δ*abcBA* and stimulated with *B*. *canis*, with no significant difference between splenocytes from mice stimulated with *B*. *ovis* or *B*. *canis* (p > 0.05). As expected, splenocytes from mice inoculated with sterile alginate capsules or PBS did not respond to stimulation with *B*. *ovis* or *B*. *canis* inactivated by gamma irradiation with IFN-γ production (Fig 2).

IL-10 was produced by splenocytes from mice immunized with alginate-encapsulated *Bo*Δ*abcBA* and stimulated with either *B*. *ovis* or *B*. *canis* although with a much lower magnitude of response (approximately 2-fold) when compared to IFN-γ. Splenocytes from mice inoculated with sterile alginate capsules or PBS did not respond to stimulation with *B*. *ovis* or *B*. *canis* inactivated by gamma irradiation with IL-10 production (Fig 2).

### *Brucella ovis* Δ*abcBA* encapsulated with alginate protects against *Brucella canis* infection

**Fig 3:**
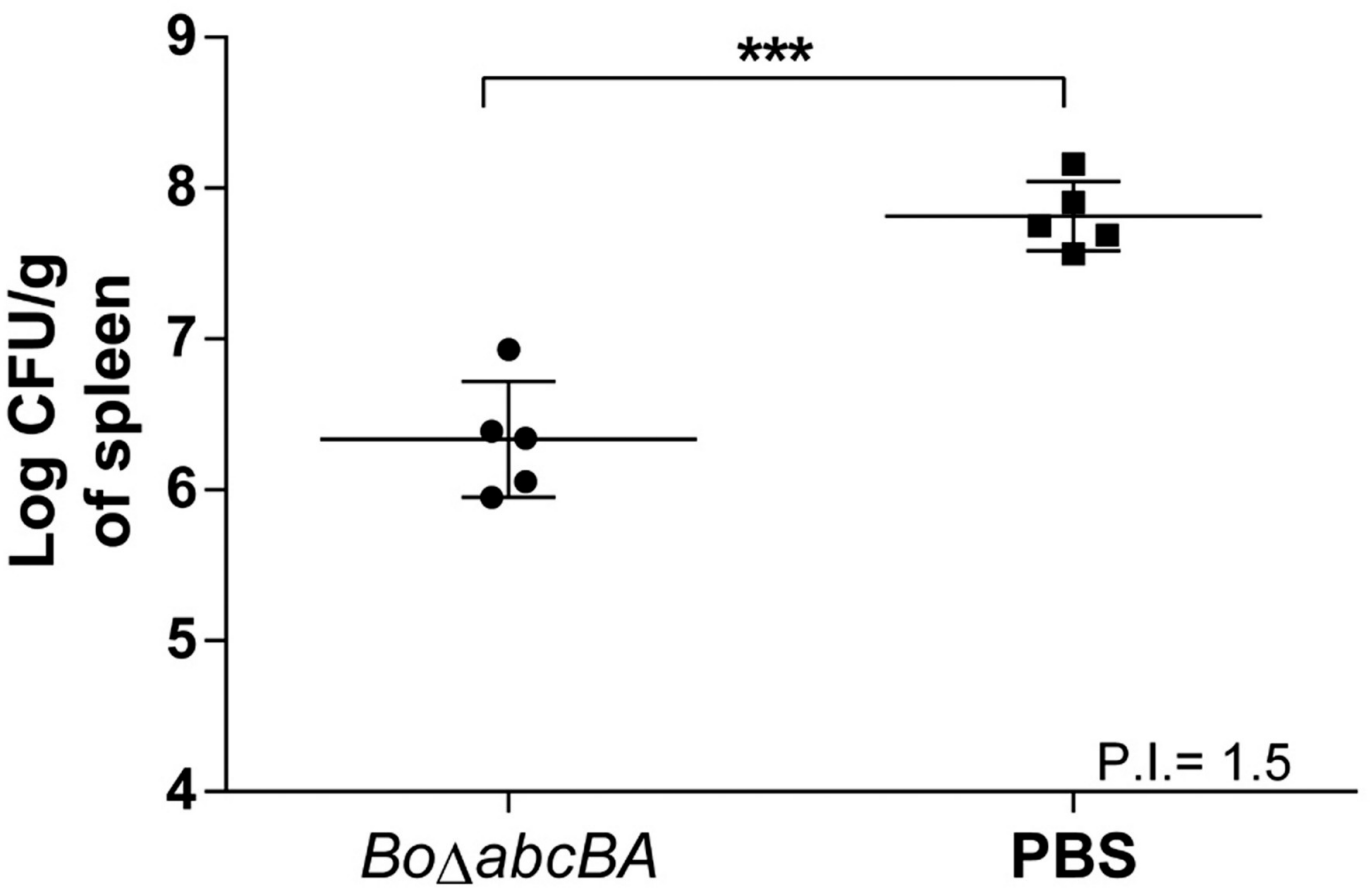
Log CFU of *Brucella canis* per gram of spleen of mice immunized with alginate-encapsulated *Brucella ovis* Δ*abcBA* (*Bo*Δ*abcBA*; n = 5) or PBS (n = 5) and, four weeks later, challenged with wild type *B*. *canis* (10^6^/mouse) intraperitoneally. Spleens were collected two weeks after challenge. Each data point represents one mouse and the bar indicates the mean of the group. Bacterial numbers were logarithmically transformed and submitted to ANOVA and means were compared using unpaired T test (***p < 0.001). P.I. (Protection Index) indicated in the figure considered the difference of CFU count in group inoculated with PBS to group immunized with alginate-encapsulated *Bo*Δ*abcBA*.

In previous studies, the strong attenuation and low persistence of *Bo*Δ*abcBA* in mice [20] was compensated by microencapsulation with alginate resulting in higher protection against challenge with WT *B*. *ovis* [29]. Therefore, in this study mice were immunized with alginate-encapsulated *Bo*Δ*abcBA* followed by challenge with WT *B*. *canis* at 4 weeks after immunization. Mice were euthanized two weeks after challenge and bacterial loads in spleen were quantified (Fig 3).

*B*. *canis* CFU numbers were significantly reduced (p < 0.001) in the spleen of mice immunized with alginate-encapsulated *Bo*Δ*abcBA* (6.3 log CFU) when compared to mice inoculated with sterile PBS (7.8 log CFU) (Fig 3). Importantly, *Bo*Δ*abcBA* was not recovered from the spleen of immunized mice. Therefore, this experimental vaccine protocol resulted in a protection index of 1.5 after challenge with *B*. *canis*.

**Fig 4:**
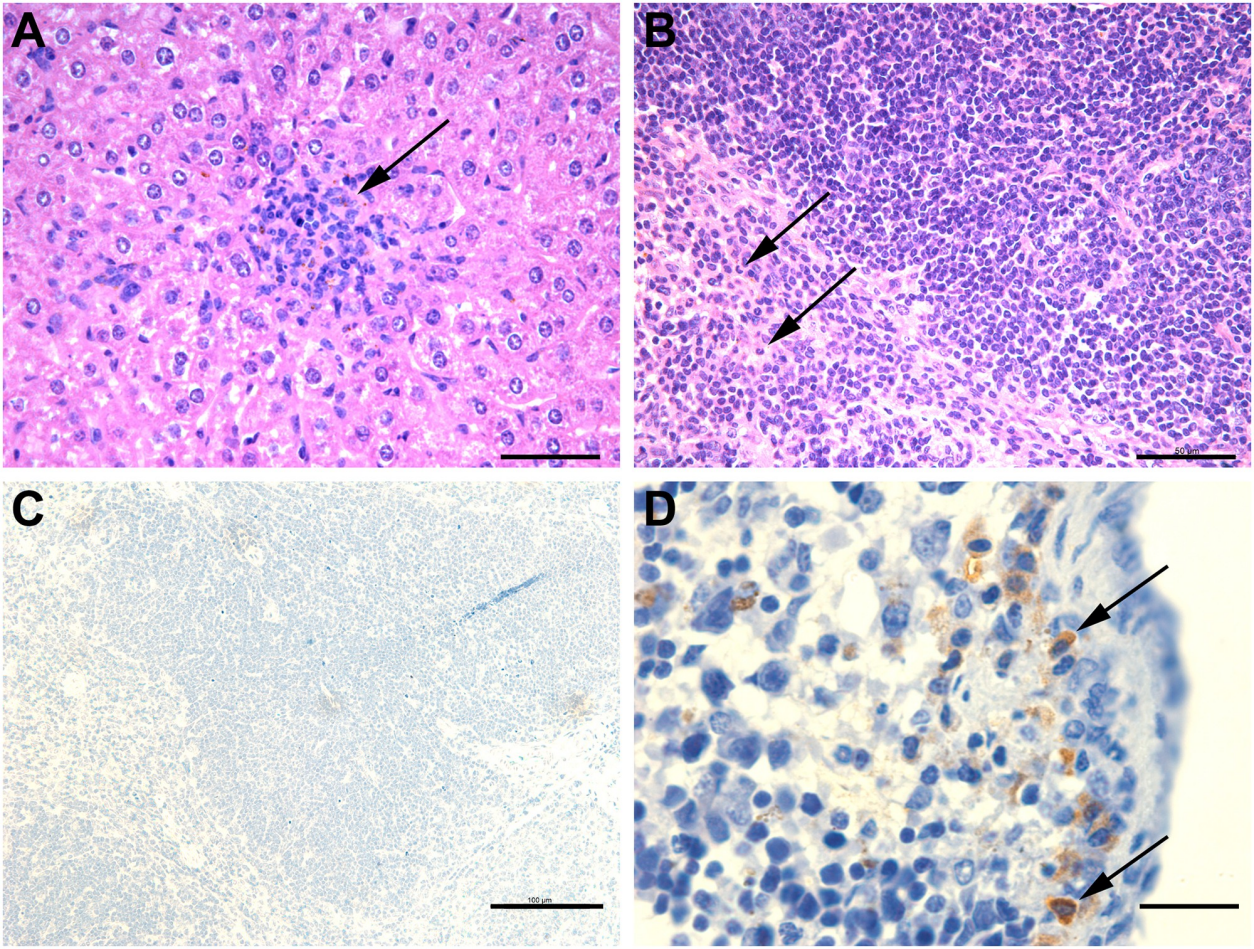
Representative microscopic changes in the liver (A) and spleen (B) of non-immunized BALB/c challenged with *Brucella canis*. Arrows indicate microgranuloma in liver and inflammatory infiltrate in spleen. Hematoxylin and eosin, Bars = 50 μm. (C) immunohistochemistry negative control. DAB (3,3'-diaminobenzidine) counterstained with Mayer’s hematoxylin, Bar = 100 μm. (D) Immunolabeling of intralesional *Brucella* sp., mostly associated with macrophages in the spleen of mice challenged with *B*. *canis*. Inset: immunohistochemistry negative control. DAB (3,3'-diaminobenzidine) counterstained with Mayer’s hematoxylin, Bar = 50 μm.

There were no gross lesions in spleen and liver of immunized and non-immunized mice. Microscopically, similar inflammatory responses were observed in liver and spleen of immunized and non-immunized mice, as demonstrated by statistically similar histopathology scores (p > 0.05). In the liver, there were mild microgranulomas with rare mild foci of necrosis (Fig 4A). In the spleen and liver, mild to moderate multifocal inflammatory infiltrate composed by neutrophils and macrophages were randomly distributed (Fig 4B). Intralesional bacteria were detected by immunohistochemistry mostly associated with macrophages (Fig 4C and 4D).

### The vaccine strain *Brucella ovis* Δ*abcBA* is safe for dogs

Considering the immune response and protection provided by the vaccine strain *Bo*Δ*abcBA* in mice, next we evaluated the safety of the vaccine strain for dogs, the natural host of *B*. *canis* and the target species for vaccination.

None of the immunized or control dogs developed any clinical sign compatible with *Brucella* sp. infection. There were no changes in the volume and consistency of lymph nodes, testicles and epididymis throughout the course of the experiment, as well as no vaginal discharges in females.

**Fig 5:**
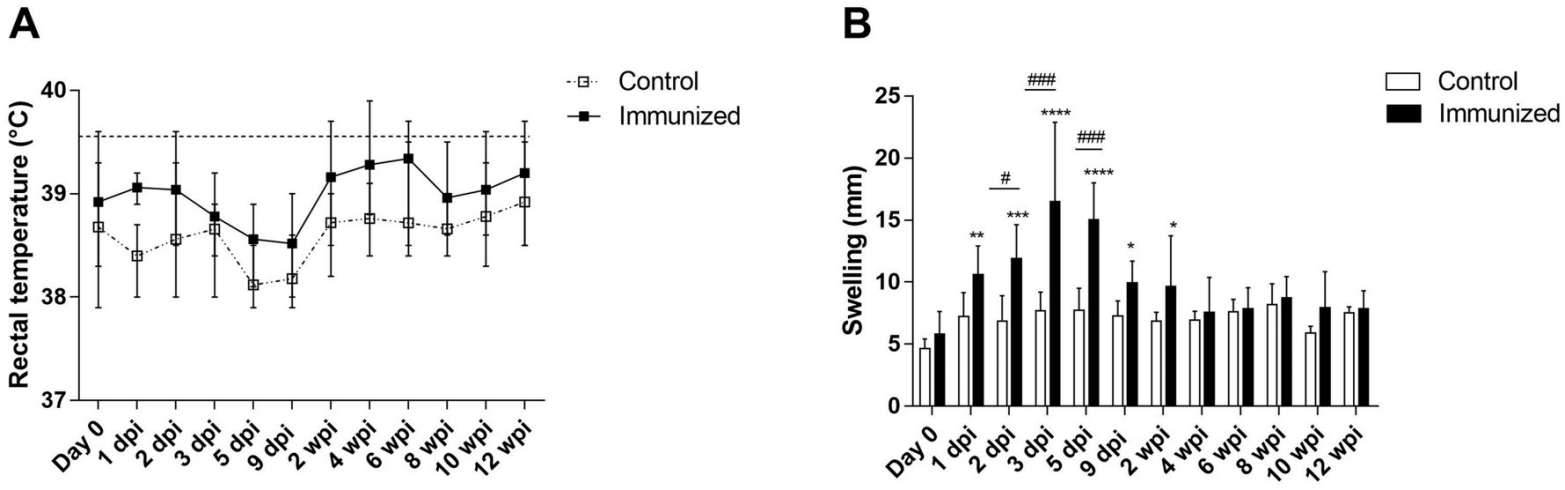
Rectal temperature (A) and swelling at the site of inoculation (B) in dogs immunized with alginate-encapsulated *B*. *ovis* Δ*abcBA* (Immunized) or inoculated with sterile alginate capsules (control). Each data point of rectal temperature represents the mean (n = 5) and the bar represent the maximum and minimum temperature. The dashed line indicates the upper limit of normal range for rectal temperature (39.5°C; Lunn, 2001). In B, columns represent mean of dogs immunized with alginate-encapsulated *B*. *ovis* Δ*abcBA* (Immunized; n = 5) or inoculated with sterile alginate capsules (Control; n = 5), and error bars indicate the standard deviation (SD). Data were submitted to ANOVA and means were compared between groups in a given time point using the Sidak’s multiple comparisons test (# = p < 0.05; ### = p < 0.001) or Dunnett’s multiple comparisons test Tukey test for comparing a given time point to day 0 (* = p < 0.05; ** = p < 0.01; *** = p < 0.001).

Fever is an important marker of inflammation in dogs. Fig 5A demonstrated the rectal temperature of control and dogs immunized with alginate-encapsulated *Bo*Δ*abcBA*. There were no significant differences in rectal temperature when immunized dogs were compared to controls. The local reaction at the site of inoculation increased up to 3 days post immunization (dpi) in dogs immunized with alginate-encapsulated *Bo*Δ*abcBA* and progressively regressed until 4 weeks post immunization (wpi) (Fig 5B). Dogs inoculated with sterile alginate capsules did not develop local reaction at the site of inoculation at any time point of the study, indicating that local reaction is promoted by the presence of the vaccine strain *Bo*Δ*abcBA*.

### Immunization did not alter hematological and biochemical profiles in dogs

**Fig 6:**
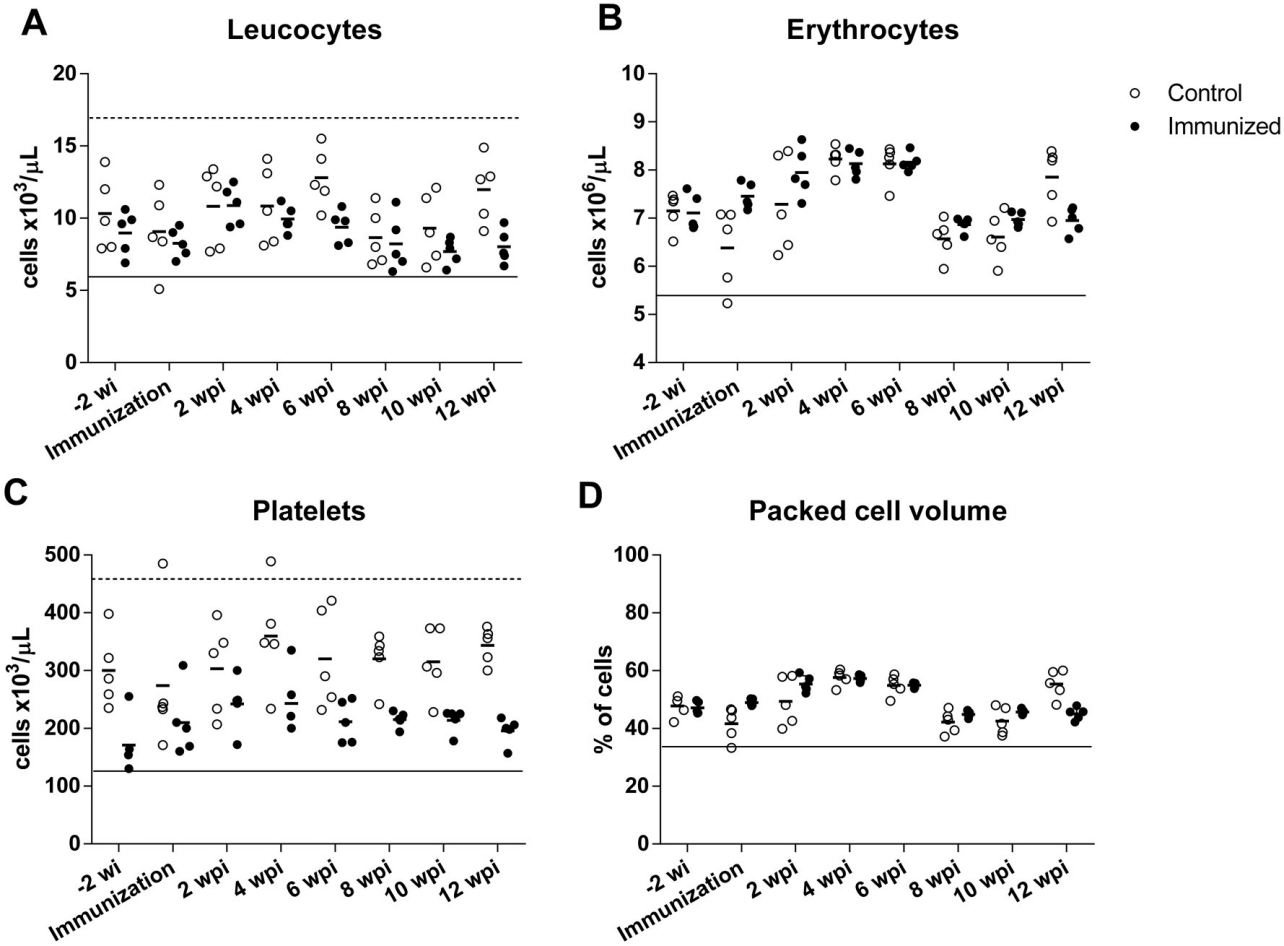
Hematological parameters of dogs immunized with alginate-encapsulated *B*. *ovis* Δ*abcBA* (immunized) or inoculated with sterile alginate capsules (control). Horizontal lines indicate the mean numbers of leucocytes (A), erythrocytes (B), platelets (C) and packed cell volume (D), and individual values are indicated by open dots (Control) or closed dots (Immunized). Reference values are indicated by a dashed line (upper limit) and a continuous line (lower limit).

There were no significant changes in hematological parameters of either control or immunized dogs at any time point (Fig 6), with values remaining within the reference range (S2 Table). Additionally, there were no significant changes in the differential count of leucocytes throughout the course of the experiment (S3 Table).

**Fig 7:**
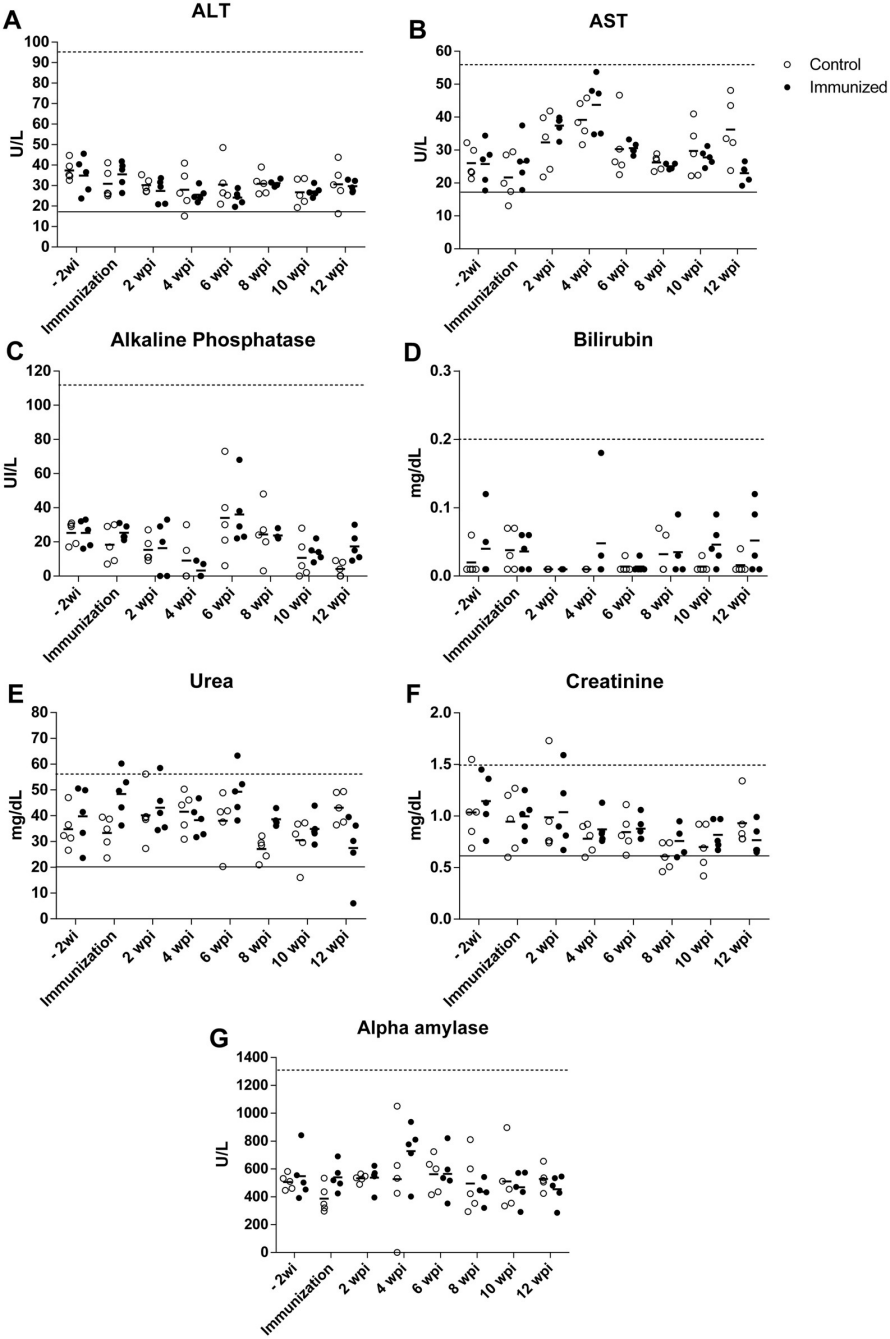
Biochemical parameters of hepatic, renal, and pancreatic functions of dogs immunized with alginate-encapsulated *B*. *ovis* Δ*abcBA* (immunized) or inoculated with sterile capsules of alginate (control). Horizontal lines indicate the mean value of ALT (alanine aminotransferase) (A), AST (aspartate aminotransferase) (B), alkaline phosphatase (C), total bilirubin (D), urea (E), creatinine (F), and alpha amylase (G) measured by colorimetric analysis. Individual values are represented by open dots for Control and closed dots for Immunized dogs. Reference values are indicated by a dashed line (S2 Table).

Biochemical profiles of control and immunized dogs are represented in Fig 7. No significant changes in ALT, AST, alkaline phosphatase, and total bilirubin were observed.

There were no significant changes in hematological and biochemical parameters of dogs in this study, without any significant differences between immunized and control dogs (p > 0.05). These results indicate that immunization with *Bo*Δ*abcBA* encapsulated with alginate, as well as alginate alone did not have any deleterious effect on hematological and biochemical parameters of immunized dogs.

### *Brucella ovis* Δ*abcBA* did not cause genital changes and it was not shed by immunized dogs

There were no genital clinical changes in immunized dogs or control animals inoculated with sterile alginate capsules.

Bacterial isolation and PCR for detection of *Bo*Δ*abcBA* was performed using blood, urine, semen and vaginal swabs every 2 wpi in immunized and control dogs. *Bo*Δ*abcBA* was not isolated or detected by PCR throughout the entire course of the experiment, indicating that bacteria is not carried in blood, and is not shed through urine, semen or vaginal secretions.

Immunized dogs were submitted to euthanasia at 22 wpi, and samples of the skin (site of inoculation), lymph nodes (retromandibular, axillar, inguinal, popliteal, subscapular, and cervical), spleen, liver, pancreas, stomach, bladder, kidney (right and left), urethra, ureter, testicles/ovary (right and left), epididymis (right and left), prostate/uterus, penis, central nervous system, heart, lung and bone marrow, were analyzed by histopathology.

There were no pathologic changes (either gross or microscopic) in immunized dogs. All tissue samples from all immunized dogs were negative for *Brucella* spp. by PCR. Interestingly, *Bo*Δ*abcBA* was isolated from subscapular and cervical lymph nodes (near the inoculation site) of two immunized dogs.

### Immunization with *Brucella ovis* Δ*abcBA* induced antibody response in dogs

Two weeks after immunization, 60% (3/5) of immunized dogs had detectable antibodies against rough *Brucella*. At 4 wpi, 80% of immunized dogs (4/5) had antibodies against rough *Brucella*. Importantly, at 4 wpi all immunized dogs have had detectable antibodies in at least one time point post immunization, indicating that all immunized dogs seroconverted (Table 1). None of the negative control dogs that were inoculated with sterile alginate developed anti-*Brucella* spp. antibodies, indicating that alginate did not interfere with this serologic analysis (Table 1).

**Table 1:** Seroconversion of dogs immunized with alginate-encapsulated *Brucella ovis* Δ*abcBA* (immunized) or inoculated with sterile alginate capsules (control) evaluated by Agar Gel Immunodiffusion (AGID) at various time points before and after immunization.

Time of immunization/Dog	Control					Immunized					P
	1	2	3	4	5	6	7	8	9	10	
**2 wbi**	-	-	-	-	-	-	-	-	-	-	ns
**Immunization**	-	-	-	-	-	-	-	-	-	-	ns
**2 wpi**	-	-	-	-	-	+	-	+	-	+	ns
**4 wpi**	-	-	-	-	-	-	+	+	+	+	*
**6 wpi**	-	-	-	-	-	-	+	+	+	+	*
**8 wpi**	-	-	-	-	-	-	+	+	+	+	*
**10 wpi**	-	-	-	-	-	-	+	+	+	+	*
**12 wpi**	-	-	-	-	-	-	+	+	+	+	*

wbi: weeks before immunization; wpi: weeks post-immunization; ns: non-significant.

* indicate statistically significant differences by the Fisher’s exact test (p < 0.05).

## Discussion

This study provides original data that lays the foundation for the development of an efficacious vaccine formulation for controlling canine brucellosis. Vaccination with RB51, S19, and REV-1 is largely used in cattle and small ruminants [35,36]. However, these vaccine strains have pathogenic potential for humans and animals, they interfere with serologic diagnosis, and they are not indicated for controlling canine brucellosis [35,37]. Considering the high conservation between *Brucella* species [38] and the low pathogenicity of *B*. *ovis* [39], we proposed a *B*. *ovis* based vaccine strategy for prevention of *B*. *canis* infection. Importantly, there are no commercially available vaccines for prevention of canine brucellosis, and considering that safety is a key feature for the development of new vaccination strategies, this study demonstrated that the candidate vaccine strain *Bo*Δ*abcBA* is protective against experimental *B*. *canis* challenge in mice and it is safe for dogs.

In contrast to livestock, the close contact of humans with dogs makes safety an even more important issue since pet owns must not be exposed to potentially zoonotic attenuated vaccine strains. Vaccine strains used for livestock, namely *B*. *abortus* RB51, *B*. *abortus* B19 and *B*. *melitensis* Rev1 are often shed through the milk and vaginal secretions of vaccinated animals, being a relevant source of infection for humans [37,40]. Notably, the parental strain used for vaccine development in this study is not considered zoonotic [27]. Furthermore, there is no evidence of *Bo*Δ*abcBA* shedding by immunized dogs, which is in good agreement with previous observations in immunized rams, the preferential host species for *B*. *ovis* that also do not shed the vaccine strain [22,25]. *Bo*Δ*abcBA* was isolated from lymph nodes adjacent to the inoculation site of two immunized dogs, which in the absence of shedding of the vaccine strain does not compromise safety, while it suggests that the vaccine strain may persist enough to allow the development of a protective immune response.

Here we demonstrated that the vaccine strain *Bo*Δ*abcBA* did not cause lesions or disease in immunized dogs, which is smilar to previous findings in mice [20] and rams [22] in which this strain also did not cause lesions. Commercial vaccines against bovine brucellosis are usually administered to young and non-pregnant females, because these vaccine strains possesses residual virulence and can cause abortion in pregnant females [37] or orchitis and epididymitis in males [41]. In contrast, *Bo*Δ*abcBA* did not induce any clinical change in both male and female immunized dogs. However, future studies should address the safety of this vaccine strain in pregnant bitches.

The immunization strategy developed in this study, which employed a *B*. *ovis* mutant strain, has numerous advantages. This species is not zoonotic, it does not cause disease in species other than its preferential host (sheep), and it has structural similarities with *B*. *canis*. In addition, previous studies in mice and sheep demonstrate a great potential of protection since the attenuated vaccine strain used in this study (*Bo*Δ*abcBA*) provides sterile immunity in rams experimentally challenged with WT *B*. *ovis* [25,28]. Importantly, this is the first study demonstrating induction of immune response and protection by *Bo*Δ*abcBA* against *B*. *canis*. Interestingly, the protection index provided by alginate-encapsulated *Bo*Δ*abcBA* against *B*. *canis* in mice (1.5) was higher than that previously demonstrated in mice against *B*. *ovis* experimental challenge [28], although immunized mice developed lesions that were similar to non-immunized controls. A protection index of 1.5 may be considered intermediate [26], the *Bo*Δ*abcBA* had a relatively low protection index against virulent *B*. *ovis* in the mouse model [29], but resulted in sterile immunity in its natural host, since experimentally challenged immunized rams did not shed the virulent *B*. *ovis* strain, did not developed clinical signs or lesions [25]. These results clearly indicate a high potential of this vaccine strain for protection of dogs against natural infection. Attenuated strains tend to confer the best protection against *Brucella* spp. [26], but this vaccine strategy has not been extensively studied for prevention of *B*. *canis* infection, with only one previous study evaluating a *B*. *canis* mutant strain in mice [42], and another study that assessed protection provided by the vaccine strain *B*. *abortus* RB51 against *B*. *canis* challenge in mice [43], although RB51 has zoonotic potential, which limits its suitability to dogs.

This is the first study that demonstrated the capacity of *Brucella* spp. other than *B*. *canis* to invade and multiply within primary canine macrophages. Infection of dogs with *B*. *abortus* [44], *B*. *suis* [45,46], or with vaccine strain Rev-1 [47] have been previously reported, suggesting that dogs may play a role disseminating brucellosis among farm animals [44]. However, the capacity of another rough *Brucella* species (such as *B*. *ovis*) to infect primary macrophages of dogs has not been previously demonstrated. Here we demonstrated that WT *B*. *ovis* invades and multiplies within canine macrophages. Conversely, WT *B*. *canis* is capable of multiplying in ovine macrophages. The vaccine strain *Bo*Δ*abcBA*, in contrast to the WT strain, is attenuated in ovine macrophage [24], and displays the same profile in canine primary macrophages. Attenuation in cells is a common feature of vaccine strains [43]. Therefore, these results support the notion that a *B*. *ovis* attenuated strain may be suitable as a vaccine candidate for *B*. *canis* infection in dogs.

The ability to invade macrophages, is important for exposing antigens, possibly inducing cross protection against other species of *Brucella* [43], due to the markedly high similarities among *Brucella* species [48]. Mice immunized with alginate-encapsulated *Bo*Δ*abcBA* responded with splenocyte proliferation and IFN-γ production in response to either *B*. *ovis* or *B*. *canis*, clearly indicating the potential of this vaccine strain to induce a cross-reactive protective response against *B*. *canis*. Encapsulation allows the slow release and prolonged exposure of mice to *Bo*Δ*abcBA* antigens, thus improving the immune response [49]. Previous studies demonstrated that alginate encapsulation improves protection provided by *Bo*Δ*abcBA* in mice challenged with WT *B*. *ovis*, whereas empty alginate capsules do not provide any protection [29].

Protective immune response to *Brucella* infection is strongly associated with IFN-γ production [36,50,51]. Thus, higher production of IFN-γ by mice immunized with alginate-encapsulated *Bo*Δ*abcBA* contributed for developing a protective immune response against *B*. *canis*. Moreover, IFN-γ is an important pro-inflammatory cytokine associated to histological lesions in mice and dogs [52]. However, production of IFN-γ after *B*. *canis* infection is lower than in response to other *Brucella* spp. [52], which is associated with milder lesions in mice challenged with *B*. *canis*, when compared to other *Brucella* spp., as observed in this study. IL-10 is considered a modulatory cytokine, acting specially to control the damage in tissues promoted by pro-inflammatory cytokines [53]. In the context of *Brucella* pathogenesis, it has been demonstrated that *B*. *abortus* is capable of inducing IL-10 production by CD4^+^CD25^+^ T cells, which impairs macrophage bactericidal activity, favoring bacterial survival and persistence of infection [54]. Although an increase of IL-10 in response to immunization as observed in this study is not unexpected since exposure to *Brucella* sp. *in vivo* triggers IL-10 production [54]. Absence of hematological and biochemical changes in immunized dogs support the notion that this experimental vaccine strain is safe for dogs, although *B*. *canis*-infected dogs may not have hematological changes even in the presence of clinical signs [52].

Recent studies aiming the development of vaccine against *B*. *canis* are mostly based on protein vaccine formulations, which tend to have limited protection in mice or in the natural hosts [55–58]. Other strategies for vaccine development against *B*. *canis* infection include lysed *B*. *abortus* [59] and *B*. *canis* non-living cells envelop lacking cytoplasmic contents [60]. Considering the potential limitations of these strategies, the use of *B*. *ovis*, which has a naturally low virulence potential for dogs and man is a promising approach for the development of an efficacious and safe vaccine formulation to prevent canine brucellosis.

## Conclusion

The alginate-encapsulated *Bo*Δ*abcBA* protects against *B*. *canis* infection in experimentally challenged mice and induced cellular immune responses. Furthermore, *Bo*Δ*abcBA* encapsulated with alginate do not promote biochemical, hematological and pathological changes, and may be considered safe for dogs.

## Supporting information

S1 Table Primer sequences used in this study. (PDF)

S2 Table Reference values for hematological parameters of adult dogs. (PDF)

S3 Table Differential count of lymphocytes, segmented cells and eosinophils in dogs immunized with *B*. *ovis* Δ*abcBA* encapsulated with alginate (immunized) or immunized with sterile capsules of alginate (control). (PDF)

S1 File The ARRIVE guidelines checklist. (PDF)
