# Supplementary material for: Brucella ovis mutant in ABC transporter protects against Brucella canis infection in mice and it is safe for dogs
Source: PLoS One. 2020 Apr 16;15(4):e0231893. doi: 10.1371/journal.pone.0231893 (PMC7162469; doi:10.1371/journal.pone.0231893)
Supplement: S1 Table — (PDF) [file pone.0231893.s001.pdf]

Brucella ovis mutant in ABC transporter protects against Brucella canis infection in mice and it is safe for dogs

Camila Eckstein, Juliana P. da Silva Mol, Fabíola Barroso Costa, Philipe P. Nunes, Pâmela A. Lima, Marília M. Melo, Thaynara P. Carvalho, Daniel O. dos Santos, Monique F. Silva, Tatiane Furtado de Carvalho, Luciana Fachini da Costa, Otoni A. O. Melo Júnior, Rodolfo C. Giunchette, Tatiane Alves Paixão, Renato Lima Santos

S1 Table. Primer sequences used in this study.

**Target**	**Sense (5'-3')**	**Anti-sense (5'-3')**	**Product**	**Reference**
Brucella spp.	TGGCTCGGTTGCCAATATCAA	CGCGCTTGCCTTTCAAGGTCTG	223 bp	60
Brucella ovis	GCCTACGCTGAAACTTGCTTTTG	ATCCCCCCATCACCATAACCGAAG	228 bp	66
Brucella ovis ΔabcBA	GGCCCGGTTTTCTGTCTCCAA	TCATCACGGTACTTGGGCTC	174 bp	31
