# Supplementary material for: Brucella ovis mutant in ABC transporter protects against Brucella canis infection in mice and it is safe for dogs
Source: PLoS One. 2020 Apr 16;15(4):e0231893. doi: 10.1371/journal.pone.0231893 (PMC7162469; doi:10.1371/journal.pone.0231893)
Supplement: S2 Table — (PDF) [file pone.0231893.s002.pdf]

Brucella ovis mutant in ABC transporter protects against _Brucella canis_ infection in mice and it is safe for dogs

Camila Eckstein, Juliana P. da Silva Mol, Fabíola Barroso Costa, Philipe P. Nunes, Pâmela A. Lima, Marília M. Melo, Thaynara P. Carvalho, Daniel O. dos Santos, Monique F. Silva, Tatiane Furtado de Carvalho, Luciana Fachini da Costa, Otoni A. O. Melo Júnior, Rodolfo C. Giunchette, Tatiane Alves Paixão, Renato Lima Santos

S2 Table. Reference values for hematological parameters of adult dogs.

**Parameter**		**Units**	**Value**
**Hematological**	**Leucocytes**	$x10^3/\mu L$	6.0-17.0
	**Erythrocytes**	$x10^6/\mu L$	5.4-7.8
	**Packed cell volume (PCV)**	%	37-54
	**Platelets**	$x10^5/\mu L$	1.6-4.3
**Biochemical**	**ALT**	U/L	17-95
	**AST**	U/L	18-56
	**Total bilirubin**	mg/dL	0-0.2
	**Alkaline phosphatase**	U/L	7-115
	**Urea**	mg/dL	20-56
	**Creatinine**	mg/dL	0.6-1.5
	**Alfa amylase**	U/L	322-1310

Reference values based on:

1. Meyer, D. J. & Harvey, J. W. Veterinary laboratory medicine: interpretation & diagnosis (Philadelphia, 2004).
2. Chemistry (Cobas). Animal Health Diagnostic Center: Cornell University, https://www.vet.cornell.edu/animal-health-diagnostic-center/laboratories/clinical-pathology/reference-intervals/chemistry; 2016 [accessed in October 2018].
3. Kaneko, J., Harvey, J. & Bruss, M. Clinical Biochemistry of Domestic Animals (California, 2008).
