# Supplementary material for: Brucella ovis mutant in ABC transporter protects against Brucella canis infection in mice and it is safe for dogs
Source: PLoS One. 2020 Apr 16;15(4):e0231893. doi: 10.1371/journal.pone.0231893 (PMC7162469; doi:10.1371/journal.pone.0231893)
Supplement: S3 Table — (PDF) [file pone.0231893.s003.pdf]

Brucella ovis mutant in ABC transporter protects against _Brucella canis_ infection in mice and it is safe for dogs

Camila Eckstein, Juliana P. da Silva Mol, Fabíola Barroso Costa, Philipe P. Nunes, Pâmela A. Lima, Marília M. Melo, Thaynara P. Carvalho, Daniel O. dos Santos, Monique F. Silva, Tatiane Furtado de Carvalho, Luciana Fachini da Costa, Otoni A. O. Melo Júnior, Rodolfo C. Giunchette, Tatiane Alves Paixão, Renato Lima Santos

S3 Table. Differential count of lymphocytes, segmented cells and eosinophils in dogs immunized with *B. ovis* Δ*abcBA* encapsulated with alginate (immunized) or immunized with sterile capsules of alginate (Control).

Sampling	Group	Lymphocyte (10^3/µL)	Segmented cell (10^3/µL)	Eosinophil (10^3/µL)
-2wi	Control	2.50±0.9	7.36±2.34	0.46±0.05
	Immunized	2.02±0.68	6.20±1.26	0.76±0.35
Immunization	Control	2.16±0.51	6.28±2.21	0.64±0.24
	Immunized	1.82±0.23	5.06±1.98	0.56±0.08
2 wpi	Control	2.50±0.37	5.68±3.70	0.64±0.13
	Immunized	2.3±0.49	8.00±1.10	0.58±0.08
4 wpi	Control	2.72±0.64	7.30±1.99	0.82±0.27
	Immunized	2.06±0.73	7.14±0.51	0.74±0.32
6 wpi	Control	2.90±0.84	9.12±1.33	0.78±0.30
	Immunized	1.96±0.49	7.00±1.00	0.42±0.08
8 wpi	Control	1.94±0.59	6.16±1.51	0.56±0.21
	Immunized	1.50±0.43	6.30±1.63	0.42±0.20
10 wpi	Control	1.90±0.56	6.92±2.04	0.50±0.18
	Immunized	1.40±0.29	5.90±0.55	0.40±0.15
12 wpi	Control	3.20±0.66	7.88±1.60	0.90±0.41
	Immunized	1.62±0.40	5.44±0.58	0.96±0.82
Reference Values*		1.0-4.8	3.0-11.5	0.1-1.25

*Reference Values according to Meyer and Harvey (2004)[43].
